# Sex-Related Differences in Regional Blood–Brain Barrier Integrity in Non-Demented Elderly Subjects

**DOI:** 10.3390/ijms22062860

**Published:** 2021-03-11

**Authors:** Yeonsil Moon, Changmok Lim, Yeahoon Kim, Won-Jin Moon

**Affiliations:** 1 Department of Neurology, Konkuk University Medical Center, Konkuk University School of Medicine, Seoul 05030, Korea; 20060246@kuh.ac.kr; 2 Department of Radiology, Konkuk University School of Medicine, Seoul 05030, Korea; changmok89@korea.kr (C.L.); yeahoon.brooke.kim@gmail.com (Y.K.); 3 Department of Radiology, Konkuk University Medical Center, Konkuk University School of Medicine, Seoul 05030, Korea

**Keywords:** blood–brain barrier, Occipital Lobe, magnetic resonance imaging, cognition, permeability, humans, female, male

## Abstract

The role of the blood–brain barrier (BBB) breakdown has been recognized as being important in Alzheimer’s disease pathogenesis. We aimed to evaluate whether regional BBB integrity differed according to sex and whether differences in BBB integrity changed as a consequence of aging or cognitive decline, using dynamic contrast-enhanced (DCE)-magnetic resonance imaging (MRI). In total, 75 participants with normal cognition (NC) or mild cognitive impairment (MCI) underwent cognitive assessments and MRI examination including DCE-MRI. Regional K_trans_ was calculated in cortical regions and the Patlak permeability model was used to calculate BBB permeability (K_trans_, min^−1^). Females had a lower median K_trans_ in the cingulate and occipital cortices. In the “older old” group, sex differences in K_trans_ were only observed in the occipital cortex. In the MCI group, sex differences in K_trans_ were only observed in the occipital cortex. Age was the only predictor of cognitive assessment scores in the male MCI group; however, educational years and K_trans_ in the occipital cortex could predict cognitive scores in the female MCI group. Our study revealed that females may have better BBB integrity in cingulate and occipital cortices. We also found that sex-related differences in BBB integrity are attenuated with aging or cognitive decline.

## 1. Introduction

Alzheimer’s disease (AD) is the most common cause of dementia and is characterized by abnormal protein deposition of amyloid-β (Aβ) and tau proteins. Besides abnormal protein deposition, the role of the blood–brain barrier (BBB) breakdown is being recognized as a major contributor to AD pathogenesis [1,2,3]. BBB is critical to normal brain functions as it regulates the entry of nutrients, ions, and other molecules into the brain and protects the brain from harm caused by toxins. As the primary protective barrier of brain, BBB exists between blood system and brain parenchyma and consists of various structural components, such as endothelial cells, astrocytes, pericytes, and tight junction proteins [4]. BBB breakdown demonstrated in APP, PSEN1, tau, and pericyte-deficient transgenic mouse models implies that Aβ pathology could promote BBB breakdown or vice versa. Other vascular-mediated diseases that are independent of Aβ and tau pathology also show early BBB breakdown [3]. BBB breakdown in AD has been confirmed in human studies [5,6,7,8,9]. Moreover, it is speculated that cerebral vascular dysfunction caused by vascular pathology might also contribute to BBB breakdown in AD [8].

Many clinical and biological variables can affect BBB integrity. Cognitive decline and the presence of the apolipoprotein E4 alle are well known factors associated with BBB integrity [3,6,8]. Many biological factors control BBB integrity, including proteins, cytokines, enzymes, and free radicals, and can be categorized to three parts: junctional proteins at the BBB, proteins of the basement membranes of the BBB, and signaling mediators [4]. Additionally, changes in the integrity and function of the BBB are common pathological mechanisms in neurodegenerative disease [10].

Although female patients are more susceptible to AD and other neurodegenerative diseases than male patients, sex differences have not been fully elucidated in terms of BBB integrity and breakdown. Sex differences in BBB integrity have been suggested in animal studies and human cerebrospinal fluid (CSF) studies. Some rodent studies have reported a protective effect of female sex hormones on BBB permeability under normal and pathological conditions. Specifically, ovariectomized rats show increased Evan’s blue dye extravasation into the brain [11] and increased myogenic tone in the brain-penetrating arteriole [12]. In one study conducted on more than 20,000 human subjects, females showed significantly lower CSF/serum albumin ratio compared to males, which highlights that females may have different level of BBB integrity (less permeable) from male counterparts [13].

To measure the integrity of the BBB, various methods such as estimating the structure of tight junctions, assessing leukocyte migration, or measuring water homeostasis can be used, but evaluating BBB permeability is the most widely applied. There are several methods for assessing BBB permeability in humans, including a direct examination of brain tissue, the CSF-albumin index, and imaging techniques. Examination of postmortem human tissue is limited because it does not reflect the temporal condition or cognitive capacity of the patients. The CSF-albumin index (the CSF/serum albumin ratio) is a standardized biomarker that reflects BBB function. However, this method also has limitations in that the lumbar puncture used to obtain the samples is very intrusive and ultimately this method cannot be used to determine the location of the BBB leakage. Moreover, as albumin is a protein molecule with a fairly large molecular weight of 66.5 kDa, it can only be measured in cases of significantly advanced BBB damage and does not reflect early changes in BBB permeability [9,14,15].

Recently, neuroimaging using dynamic contrast-enhanced (DCE)-magnetic resonance imaging (MRI) has been suggested as an attractive alternative method for assessing regional BBB permeability. This reflects the temporal state of BBB permeability in humans, via non-invasive methods. Moreover, by using neuroimaging analysis, it is possible to reveal discrete regional changes [3,6,8,9,16]. A few DCE-MRI studies of mild cognitive impairment (MCI) and AD have reported increased BBB permeability in the hippocampus and several gray and white matter regions, before the onset of brain atrophy or dementia [8], and have made associations between BBB permeability and cognitive decline [6,7,8,9]. MCI corresponds to an intermediate stage in the extension line of cognitive function that connects normal and dementia, and the basic concept is “a state where cognitive decline is more severe than expected, but not dementia”. The distinction between MCI and dementia is determined by whether cognitive decline is severe enough to lead to functional impairment of daily living. One of the most common causes of MCI is AD, which is also most common cause of dementia. AD is a neurodegenerative disease characterized by loss of neurons and synapses in the cerebral cortex and certain subcortical regions.

However, detailed analyses of precise anatomical distribution are rare [6].

Hence, we aimed to evaluate whether regional BBB integrity differs according to sex and whether the sex-differences in BBB integrity change according to clinical status, as assessed via the DCE-MRI-based BBB permeability imaging technique.

## 2. Results

### 2.1. Demographic Characteristics

The mean age of the participants was 67.1 years old, and 24 patients were male and 51 were female. When stratified according to cognitive status, there were 36 patients in the normal cognition (NC) group and 39 in the MCI group. Males and females did not differ significantly with respect to age, the ratio of MCI, and scores on the Mini-Mental Status Examination (MMSE) or the Clinical Dementia Rating Scale-Sum of Boxes (CDR-SOB) assessment. Only “educational years” were lower in the female group (9.9 ± 3.7 vs. 12.9 ± 4.2 years) versus males. The cortical volume ratio was generally higher in female than in male in all cortical regions except the occipital cortex (Table S1). The demographic characteristics of each group are summarized in Table 1.

### 2.2. Comparison of Cerebral Regional BBB Permeability

In regression analysis, we found that any of vascular risk factors had an effect on the K_trans_ of all cortical regions. The median K_trans_ of white matter is related with K_trans_ of all cortical regions.

The K_trans_ of the six cortical regions were also related to each other; however, K_trans_ of the occipital cortex was less related to those of the frontal and insular cortex (Table 2).

**Figure 1:**
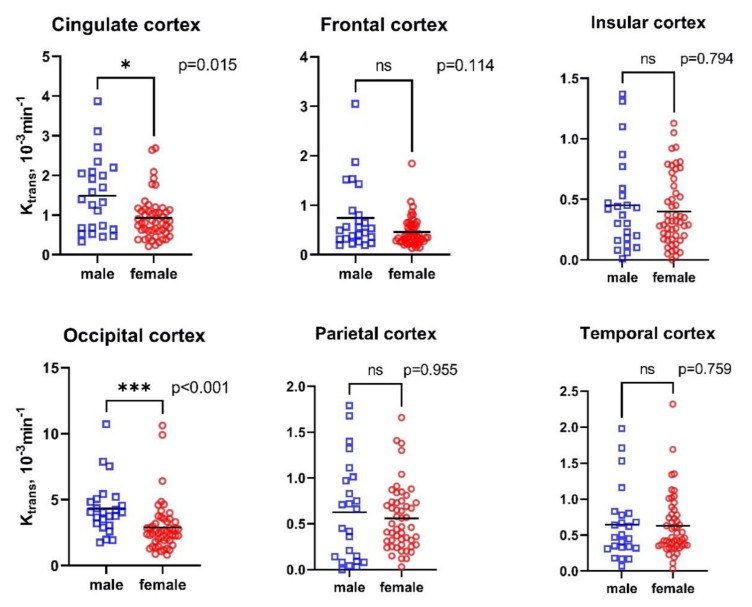
Differences in cerebral regional K_trans_ according to sex. *p*, *p*-value; *, *p*-value < 0.05; ***, *p*-value < 0.001.

Compared with males, females had lower median K_trans_ in the cingulate (median 0.81 × 10^−3^ min^−1^, min–max 0.21–2.69 × 10^−3^ min^−1^ vs. median 1.36 × 10^−3^ min^−1^, min–max 0.33–3.87 × 10^−3^ min^−1^, *p*-value = 0.015) and occipital cortices (median 2.57 × 10^−3^ min^−1^, min–max 0.81–10.62 × 10^−3^ min^−1^ vs. median 4.03 × 10^−3^ min^−1^, min–max 1.74–10.72 × 10^−3^ min^−1^, *p*-value < 0.001, Figure 1).

**Figure 2:**
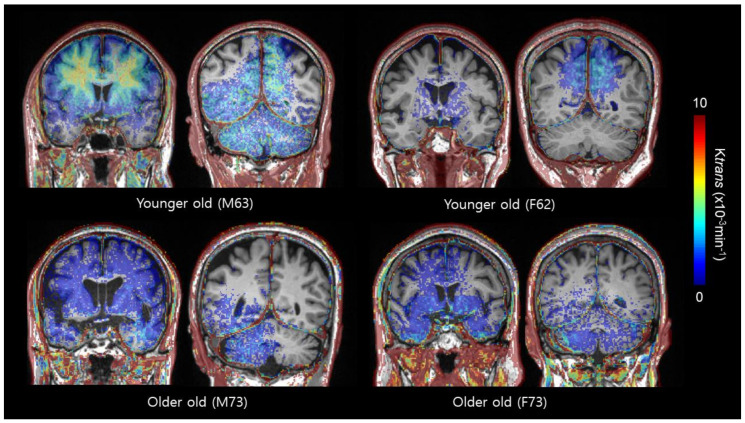
Exemplary cases of BBB permeability imaging (K_trans_) by sex and age group. Higher BBB permeability in the cingulate and occipital cortex is noted in male subjects compared to female subjects.

As the mean age of participants was 67.1 years old and the median value was 68 years old, we classified “younger old” as individuals who were 67 and below, and those who were above 67 were classified as “older old”. We then evaluated how sex differences in K_trans_ were changed as a function of age. No significant differences in the ratio of MCI (*p*-value = 0.641 and 0.578), MMSE (*p*-value = 0.086 and 0.989), or CDR-SOB scores (*p*-value = 1.00 and 0.110) were observed between females and males in either the “younger old” or “older old” groups. In the “younger old” group, the median K_trans_ was lower in the cingulate (median 0.85 × 10^−3^ min^−1^, min–max 0.21–2.69 × 10^−3^ min^−1^ vs. median 1.70 × 10^−3^ min^−1^, min–max 0.33–3.87 × 10^−3^ min^−1^, *p*-value = 0.005), and occipital cortices (median 2.47 × 10^−3^ min^−1^, min–max 0.88–9.91 × 10^−3^ min^−1^ vs. median 4.02 × 10^−3^ min^−1^, min–max 1.93–5.42 × 10^−3^ min^−1^, *p*-value = 0.012) of the female group compared with the male group. In the “older old” group, sex differences in K_trans_ were only observed in the occipital cortex (median 0.81 × 10^−3^ min^−1^, min–max 0.81–10.62 × 10^−3^ min^−1^ vs. median 4.05 × 10^−3^ min^−1^, min–max 1.74–10.72 × 10^−3^ min^−1^, *p*-value = 0.011, Table 3 and Figure 2).

**Figure 3:**
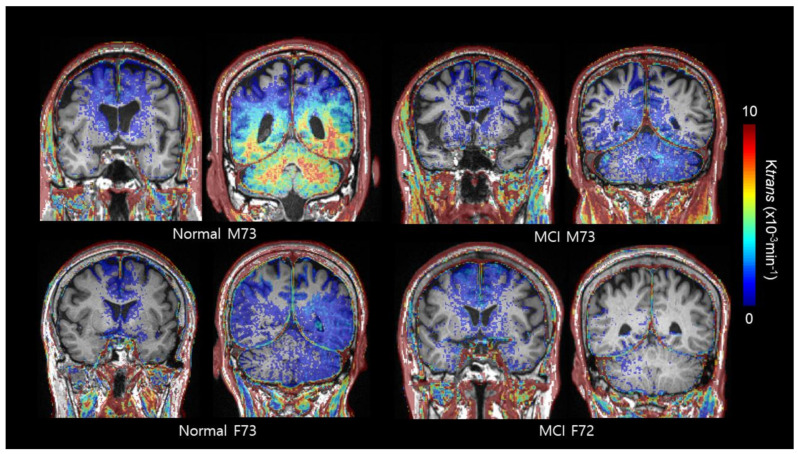
Exemplary cases of BBB permeability imaging (K_trans_) by sex and cognitive status. In the normal cognition group, high er BBB permeability in the cingulate and occipital cortex is noted in male subjects compared to female subjects. In contrast, sex differences in K_trans_ were only observed in the occipital cortex in the mild cognitive impairment (MCI) group.

When classifying the participants according to their cognitive status, neither age nor cognitive score was different as a function of age stratification (*p*-value = 0.387 and 0.0.598, respectively), MMSE (*p*-value = 0.667 and 0.558, respectively), or CDR-SOB (*p*-value = 0.641 and 0.449, respectively) scores, nor was any difference observed between females and males in both the NC and MCI groups. In the NC group, median K_trans_ were lower in the cingulate (median 0.73 × 10^−3^ min^−1^, min–max 0.24–2.69 × 10^−3^ min^−1^ vs. median 1.57 × 10^−3^ min^−1^, min–max 0.45–3.87 × 10^−3^ min^−1^, *p*-value = 0.019), frontal (median 0.33 × 10^−3^ min^−1^, min–max 0.13–1.07 × 10^−3^ min^−1^ vs. median 0.49 × 10^−3^ min^−1^, min–max 0.26–3.05 × 10^−3^ min^−1^, *p*-value = 0.047), and occipital cortices (median 2.30, × 10^−3^ min^−1^ min–max 0.81–9.91 × 10^−3^ min^−1^ vs. median 4.82 × 10^−3^ min^−1^, min–max 1.74–7.87 × 10^−3^ min^−1^, *p*-value = 0.009) of the female group compared with the male group. In the MCI group, sex differences in K_trans_ were only observed in the occipital cortex (median 2.78 × 10^−3^ min^−1^, min–max 1.18–10.62 × 10^−3^ min^−1^ vs. median 3.74 × 10^−3^ min^−1^, min–max 1.95–10.72 × 10^−3^ min^−1^, *p*-value = 0.019, Table 4 and Figure 3).

### 2.3. Correlation between BBB Permeability and Cognitive Functioning Score

In the male MCI group, only age (B = −0.257, *p*-value = 0.004), was a predictor of MMSE score; however, in the female MCI group, educational years (B = 0.292, *p*-value = 0.001) and K_trans_ of the occipital cortex (B = −0.397, *p*-value = 0.025) could predict MMSE score. In MCI patients, only age (B = 0.052, *p*-value = 0.024) was able to predict CDR-SOB scores (Table 5).

## 3. Discussion

Our study revealed that females might have better BBB integrity in cingulate and occipital cortices compared to males. We also found that this sex-related difference in BBB integrity is attenuated as aging or cognitive decline occurs, but the difference remains in the occipital cortex regardless of these two factors. Additionally, it is noteworthy that the BBB region that affected cognition was different between males and females.

In this study, a higher K_trans_ was observed in the occipital cortex as compared to the other cortices, in both males and females. The occipital cortex is a major part of the posterior circulation system supplied by the vertebrobasilar artery, unlike the frontoparietal cortex, which is supplied by the carotid artery system (anterior circulation). It appears that a relative lack of sympathetic innervation of the posterior circulation promotes vulnerability or diminished cerebral autoregulation, and ultimately causes increased BBB leakage [17].

Furthermore, we demonstrated that the sex difference in K_trans_ was significant in the cingulate and occipital cortices. Specifically, K_trans_ was remarkably high in males, suggesting a relative increase in BBB permeability compared to females. Our finding is in line with previous work, suggesting a higher CSF/serum albumin index in male subjects compared with female subjects [13]. The protective effects of female sex hormones on BBB permeability are highlighted in some in vitro [18] and rodent [10,11,19,20] studies as well. A study conducted by Luisa Torres et al. revealed that not only the level of endothelial cells transporters expressed on the brain from the healthy female mice is higher than that of male mice, but also in vitro treatment of brain endothelial cells induce greater intracellular accumulation of the endothelial cells transporters in male mice compared to those from female mice [20]. Our finding suggests that the sex-dependent difference in BBB integrity also shows a regional difference. This regional difference might be due to underlying differences within the vascular microstructure that constitutes the BBB and is related to biological sex factors, such as estrogen receptors present at the surface of endothelial cells, differences in hematocrit, cerebral metabolism, or cerebral vascular resistance [16,18,21,22].

Data explaining why the sex difference in K_trans_ is distinct in the occipital cortex are lacking; however, two explanations are possible: First, BBB integrity might be directly affected by sex-dependent regional distribution difference of sex hormone receptors and/or sex hormones in the brain. Occipital cortex is the functional center of visual perception and visual information processing. Visual cortex is highly concentrated with estrogen producing neurons and estrogen sensitive (estrogen-receptor positive) neurons in mice study [23]. Second, the cerebral blood flow (CBF) in males might be lower than that in females, especially within the occipital cortex. There are many findings consistent with females showing higher CBF than males at most ages [10,22]. Insufficient CBF plays a significant role in attenuating the function of the BBB, as well as reducing the clearance of toxic substances and enhancing neuroinflammation.

As aging or cognitive decline occurs, the sex differences in K_trans_ within the cingulate cortex and frontal cortex were minimized. This could be interpreted as a disruption in permeability that accompanies aging or underlying pathologic conditions, such as cognitive decline, and we noted that the damage is more prominent in females compared with males. We propose that it is possible that females lose the advantage of female sex hormones with aging. Indeed, estrogen is known to have a neuroprotective effect through several mechanisms [10,11,19,21]. Age-related decline in estrogen levels or alterations in estrogen receptors may contribute to the disruption of BBB integrity [19]. Nevertheless, the persistent difference in K_trans_ within the occipital cortex may be due to the regional diminution of the protective effects of female hormone singling, being lesser applied due to the posterior circulation. However, it is more likely that the baseline differences were so pronounced and subsequently occluded any statistically significant change.

The cerebral region predicting cognitive state also differed between males and females. Our results suggesting that K_trans_ within the occipital cortex was a valid variable predicting MMSE score, only in females, is interesting. Higher K_trans_ values and higher BBB permeability were correlated to lower MMSE scores. Females tend to rely more on visual areas to accomplish the tasks [24], and visual functions are mainly mediated by the occipital cortex. According to the principle of neurovascular coupling, blood flow should be more abundant to allow for more efficient cognitive functioning. However, a lack of blood flow due to increased permeability of the BBB may hinder organized cognitive function. Unlike the MMSE scores, K_trans_ within any region did not affect CDR-SOB scores in either males or females. While the MMSE assesses only cognition, the CDRS-OB evaluates global cognition and daily life activities, which require more complex cognitive organization, as well as primary motor and sensory functions [25].

Since BBB permeability is related to microvascular injury, it was thought that vascular risk factors would affect it. However, in this study, it was found that the presence of hypertension, diabetes mellitus, and dyslipidemia did not affect BBB permeability.

There are some limitations to our study. First, the relatively small sample size may have affected the results. The number of participate is not enough to investigate the impact of sex and it could have statistical problems. However, to overcome this, statistics are presented as both numbers and figures, and, when confirmed through the figures, we think it is highly likely to be a real trend, not an error in statistics. Although the present study has a small number of participants, we think that it can play a role as an introductory study that can help determine the pathophysiology of AD or plan a drug treatment strategy in the future. Secondly, our DCE imaging acquisition time was only 10 min, which is relatively short compared to recent recommendations [26,27]. Slow subtle BBB leak could be better visualized using a modified DCE protocol with a longer acquisition time (>16 min) [26]. However, in terms of clinical practice, 10 min appears to be the maximum acquisition time which limits motion in elderly patients.

## 4. Materials and Methods

### 4.1. Participants

We recruited patients with or without subjective memory complaints who visited the department of neurology of the Konkuk University Medical Center in South Korea between June 2017 and May 2019. We assessed basic demographic characteristics including age, sex, and years of education and vascular risk factors such as hypertension, diabetes mellitus, and dyslipidemia. Comprehensive neuropsychological tests, global cognitive assessments (CDR-SOB and MMSE scores), and brain imaging were also performed. The following symptoms/diagnoses were sufficient for patient exclusion: seizures, Parkinson’s disease, multiple sclerosis, cerebral palsy, Huntington’s disease, encephalitis, vascular surgery of the brain, cancer (diagnosed within the previous three years excluding skin cancer), shortness of breath while sitting still, use of oxygen at home, kidney dialysis, liver disease, hospitalization for mental or emotional reasons (within the previous five years), drug abuse (within the previous five years), episode(s) of unconsciousness exceeding 1 h, illness resulting in a permanent decrease in memory or other mental functioning, vision impairment that would prevent reading ordinary print (even with glasses), and significant gait/mobility difficulties.

In total, 75 participants with a diagnosis of NC or MCI were included in this study. NC was diagnosed if the participants had no complaints of cognitive deterioration and none of the objective neuropsychological metrics fell below the 1.0 standard deviation, among five cognitive domains. MCI was based on the criteria suggested by Peterson et al. [28].

### 4.2. Materials

#### 4.2.1. Cognition

Comprehensive neuropsychological tests assessed the five cognitive domains attention, memory, language, visuospatial function, and frontal/executive function and were used to determine whether the participants had any objective cognitive impairment compared to the normal Korean value for the same years of education and age [29]. CDR-SOB and MMSE scores were obtained through standard procedures administered to the participant and a knowledgeable informant. MMSE is a test that briefly evaluates cognitive function and is the most widely used tool in the world for screening of dementia. It consists of 19 items. Clinical Dementia Rating (CDR) is a representative rating scale that measures the overall degree of cognitive and social functioning in dementia patients. The CDR is a standard for suggesting the degree of dementia in clinical studies in each country and is most widely used as a standard for evaluating the efficacy of dementia drugs in clinical trials. The CDR is structured to evaluate six sub-categories of memory, intelligence, judgment and problem-solving skills, social activities, family life and hobbies, and hygiene and grooming in order to evenly evaluate cognitive and social functional areas that decline in dementia. The CDR-SOB is a simple sum of the scores obtained in each of the six domains rated and provides additional information to the CDR global score in mild dementia [30].

#### 4.2.2. MRI Acquisition

MRI was performed at the Konkuk University Medical Center using a Magnetom Skyra 3.0 Tesla unit (Siemens Medical Systems, Erlangen, Germany) with a 20-channel high-resolution head coil. The MRI protocol included three-dimensional (3D) T1-weighted images, 3D fluid attenuated inversion recovery (FLAIR) images, 3D susceptibility-weighted images (SWI), and coronal DCE imaging, with a 10 min acquisition period and 10 s resolution, using Gadobutrol 1.0 mmol/kg body weight. The specific parameters and protocols used for the structural MRI and DCE-MRI are provided in Table S2. Our DCE-MRI protocol was based on previously recommend parameters [27] and validated by revealing reliable and reasonable clinical results [9].

#### 4.2.3. MRI Analysis

Nordic ICE software (Version 4.1.3) was used to process the DCE imaging data and regions of interest (ROIs) were selected by a suitably trained neuroimaging research personnel with 3 years of relevant experience, under the supervision of an expert neuroradiologist who was blinded to the clinical information. Structural imaging was facilitated by 3D T1-volume imaging. We used the Patlak model, as it is considered optimal for low-leakage conditions [31,32]. Vascular input function was obtained semi-automatically from the superior sagittal sinus using Nordic ICE software. We calculated K_trans_, which indicates the permeability of the surface area product, and is equivalent to the volume transfer constant between plasma and the extravascular extracellular space. The quantity leaking per time unit, per unit capillary plasma (min^−1^), was equivalent to 100× of mL/100 g/min.

First, automatic segmentation of distinct brain regions was performed using the InBrain (https://ww.inbrain.co.kr/ (accessed on 15 June 2017), MIDAS Information Technology Co., Ltd.Seongnam-si, Gyeonggi-do, Republic of Korea) platform. Based on Freesurfer 6.0, InBrain applies deep-learning algorithms to the analysis of failure prediction, brain extraction, white matter segmentation, and analysis quality management. As previously described, the volume of regional brain structures and white matter was extracted based on the Desikan Killiany atlas and subcortical atlases [33,34]. To correct for differences in the head size, the total grey matter volume and each cortical volume were divided by the intracranial volume. The volumes of interests (VOIs) of the cerebral cortices were co-registered to the BBB permeability map, to extract the values by using a mutual information-based algorithm, to search for an optimal rigid transformation on Nordic ICE. The VOIs included the cingulate cortex, frontal cortex, insular cortex, occipital cortex, parietal cortex, and temporal cortex.

#### 4.2.4. Statistical Methods

As all radiological variables were not normally distributed as indicated by the Kolmogorov–Smirnov test, comparisons of radiological features were assessed by the Mann–Whitney U test and Fisher’s exact test, for continuous and categorical variables, respectively. Associations between cognition and K_trans_ were evaluated with Spearman correlation analysis. The stepwise method of multivariate linear regression analysis was used to identify predictive factors for MMSE and CDR-SOB scores. SPSS (v. 17.0, SPSS Inc., Chicago, IL, USA) was used for statistical analyses, and a *p*-value < 0.05 was considered the threshold of significance.

#### 4.2.5. Protocol Approvals, Registrations, and Patient Consent

This prospective study was approved by the Institutional Review Board of the Konkuk University Medical Center (No. KUH1140118). All procedures of this study were carried out according to the ethical standards set by the Helsinki Declaration of 1975 (and as revised in 1983). Written informed consent was obtained from all participants.

## 5. Conclusions

Our results suggest that females are protected from BBB disruption that occurs during the early stages of the neurodegeneration process. BBB integrity of the occipital cortex is more vulnerable than that of other regions; however, in females, there seems to be a sex-related protective effect in most other regions of interest. Additionally, alterations in BBB integrity seem to more directly affect cognition in females. In this study, the potential underlying pathological mechanism was not investigated, however sex hormones that affect BBB structure seem to play an important role. This difference varies according to brain region and clinical status, which suggests that the regions primarily affected by the progression of neurodegenerative disease are varied. Therefore, to promote more effective drug development or to better implement interventions against BBB breakdown, sex- and region-specific strategies are suggested for future studies.

## Figures and Tables

**Table 1:** Demographic characteristics of the participants and differences according to the sex.

	Total	Male	Female	*p*-Value
		(N = 24)	(N = 51)	
Age	67.1 ± 7.4	68.4 ± 8.3	66.5 ± 6.9	0.293
Education	10.8 ± 4.1	12.9 ± 4.2	9.9 ± 3.7	0.002
Hypertension	14 (18.7%)	7 (29.2%)	7 (13.7%)	0.112
Diabetes mellitus	32 (42.7%)	10 (41.7%)	22 (43.1%)	0.905
Dyslipidemia	36 (48.0%)	9 (37.5%)	27 (52.9%)	0.215
Diagnosis				0.317
-NC	36 (48.0%)	9 (37.5%)	27 (52.9%)	
-MCI	39 (52.0%)	15 (62.5%)	24 (47.1%)	
MMSE	26.6 ± 3.0	26.5 ± 3.7	26.6 ± 2.6	0.881
CDRSOB	0.9 ± 0.8	1.1 ± 0.9	0.7 ± 0.7	0.095

NC, normal cognition; MCI, mild cognitive impairment; MMSE, mini-mental status examination; CDR-SOB, Clinical dementia rating sum of boxes.

**Table 2:** Relation of BBB permeability between white matter and cerebral regional cortex.

	Cingulate Cortex	Frontal Cortex	Insular Cortex	Occipital Cortex	Parietal Cortex	Temporal Cortex
White matter	r = 0.777	r = 0.789	r = 0.693	r = 0.273	r = 0.281	r = 0.469
	*p* < 0.001	*p* < 0.001	*p* < 0.001	*p* = 0.018	*p* = 0.014	*p* < 0.001
Cingulate cortex		r = 0.883	r = 0.672	r = 0.486	r = 0.514	r = 0.578
		*p* < 0.001	*p* < 0.001	*p* < 0.001	*p* < 0.001	*p* < 0.001
Frontal cortex			r = 0.765	r = 0.179	r = 0.429	r = 0.425
			*p* < 0.001	*p* = 0.125	*p* < 0.001	*p* < 0.001
Insular cortex				r = 0.195	r = 0.483	r = 0.667
				*p* = 0.093	*p* < 0.001	*p* < 0.001
Occipital cortex					r = 0.520	r = 0.681
					*p* < 0.001	*p* < 0.001
Parietal cortex						r = 0.651
						*p* < 0.001

r, Pearson correlation coefficient; *p*, *p*-value.

**Table 3:** Comparison of cerebral regional K_trans_ according to the sex, when stratified by age.

	Younger Old (47–67, N = 36)					Older Old (68–80, N = 39)				
	Male (N = 9)		Female (N = 27)		*p*	Male (N = 15)		Female (N = 24)		*p*
**MCI**	N = 4	44.4%	N = 9	33.3%	0.641	N = 11	73.3%	N = 15	62.5%	0.578
**MMSE**	28.67	1.65	27.52	1.92	0.086	25.20	4.05	25.63	2.93	0.989
**CDRSB**	0.55	0.52	0.63	0.71	1.000	1.40	0.98	0.87	0.76	0.110
	**median**	**Min–max**	**median**	**Min–max**		**median**	**Min–max**	**median**	**Min–max**	
**Cingulate cortex**	1.70	0.33–3.87	0.85	0.21–2.69	0.005	0.73	0.45–3.11	0.73	0.31–2.64	0.270
Frontal cortex	0.55	0.19–3.05	0.35	0.13–1.07	0.073	0.37	0.19–1.52	0.37	0.14–1.84	0.638
Insular cortex	0.53	0.18–1.37	0.36	0.00–1.13	0.205	0.29	0.01–1.31	0.28	0.05–1.05	0.853
**Occipital cortex**	4.02	1.93–5.42	2.47	0.88–9.91	0.012	4.05	1.74–10.72	2.66	0.81–10.62	0.011
Parietal cortex	0.83	0.00–1.79	0.56	0.03–1.41	0.387	0.42	0.03–1.68	0.40	0.12–1.66	0.618
Temporal cortex	0.66	0.17–1.16	0.52	0.03–2.32	0.279	0.34	0.07–1.98	0.46	0.19–1.69	0.212

*p*, *p*-value.

**Table 4:** Comparison of cerebral regional K_trans_ according to the sex when classified by cognitive status.

	Normal Cognition (N = 36)					Mild Cognitive Impairment (N = 39)				
	Male (N = 9)		Female (N = 27)		*p*	Male (N = 15)		Female (N = 24)		*p*
**Age**	66.22	6.47	63.96	5.25	0.387	69.73	9.13	69.33	7.47	0.598
**MMSE**	28.22	1.48	27.93	1.68	0.667	25.47	4.30	25.17	2.71	0.558
**CDRSB**	0.38	0.48	0.27	0.34	0.641	1.50	0.88	1.27	0.72	0.449
	**median**	**Min–max**	**median**	**Min–max**		**median**	**Min–max**	**median**	**Min–max**	
**Cingulate cortex**	1.57	0.45–3.87	0.73	0.24–2.69	0.019	1.10	0.33–3.11	1.03	0.21–2.64	0.283
**Frontal cortex**	0.49	0.26–3.05	0.33	0.13–1.07	0.047	0.55	0.19–1.87	0.43	0.14–1.84	0.558
Insular cortex	0.46	0.01–1.37	0.27	0.03–1.13	0.205	0.29	0.06–1.10	0.36	0.00–1.05	0.521
**Occipital cortex**	4.82	1.74–7.87	2.30	0.81–9.91	0.009	3.74	1.95–10.72	2.78	1.18–10.62	0.019
Parietal cortex	0.83	0.03–1.79	0.49	0.12–1.41	0.086	0.36	0.00–1.40	0.43	0.03–1.66	0.223
Temporal cortex	0.66	0.07–7.98	0.41	0.11–2.32	0.180	0.40	0.17–1.53	0.63	0.03–1.69	0.123

*p*, *p*-value.

**Table 5:** Predictors of MMSE and CDR-SOB scores in the MCI group.

	Predictors of MMSE						Predictors of CDR-SOB					
	Male			Female			Male			Female		
Stepwise	B	SE	*p*-value	B	SE	*p*-value	B	SE	*p*-value	B	SE	*p*-value
constant	44.085	5.447	<0.001	24.897	1.117	<0.001	−2.450	1.465	0.109			
Age	−0.257	0.079	0.004				0.052	0.021	0.024			
Educational years				0.292	0.086	0.001						
Cingulate cortex												
Frontal cortex												
Insular cortex												
Occipital cortex				−0.397	0.171	0.025						
Parietal cortex												
Temporal cortex												

## Data Availability

Data are available only on request due to ethical problems. The data presented in this study are available on request from the corresponding author. The data are not publicly available due to ethical problems.

## Supplementary Materials

The following are available online at https://www.mdpi.com/1422-0067/22/6/2860/s1. Please refer to Tables S1 and S2 for additional information.
