# Supplementary material for: Sex-Related Differences in Regional Blood–Brain Barrier Integrity in Non-Demented Elderly Subjects
Source: Int J Mol Sci. 2021 Mar 11;22(6):2860. doi: 10.3390/ijms22062860 (PMC8001339; doi:10.3390/ijms22062860)
Supplement: Supplementary file 1 [file ijms-22-02860-s001.pdf]

Supplementary Tables and Figures

Supplementary Table S1. Volume ratio of each cortical regions

	**Total**	**Male**	**Female**	***p*-value**
**VR of Cingulate cortex**	5.35 ± 0.58	5.14 ± 0.64	5.44 ± 0.53	0.038
**VR of Frontal cortex**	36.63 ± 3.16	34.96 ± 3.18	37.42 ± 2.84	0.001
**VR of Insular cortex**	4.35 ± 0.37	4.19 ± 0.39	4.43 ± 0.33	0.007
**VR of Occipital cortex**	1.66 ± 0.66	1.54 ± 0.62	1.72 ± 0.68	0.280
**VR of Parietal cortex**	16.05 ± 2.64	14.79 ± 3.14	16.64 ± 2.15	0.004
**VR of Temporal cortex**	29.69 ± 2.50	28.55 ± 2.44	30.23 ± 2.37	0.006

VR: volume ratio which is calculated by volume of each cortical region divided by intracranial volume.

Supplementary Tables and Figures

Supplementary Table S2. MR protocol details.

	3D MPRAGE	3D FLAIR	3D SWI	DCE-MRI
**TR (ms)**	2300	5000	29	3.1
**TE (ms)**	2.98	393	20	1.04
**TI (ms)**	900	1800		
**Flip Angle (°)**	9		15	10
**FOV (mm)**	256×256	256×256	220	225×240
**Slice thickness (mm)**	1	1	2	3
**Matrix**	256×256	256×256	512×512	180×192
**Voxel size**	1×1×1	1×1×1	0.43×0.43×2	1.25×1.25×3
**NEX**	1	1	1	
**GRAPPA factor**	2	2	2	
**Other features**				60 dynamics, 10 s temporal resolution

3D MPRAGE: three-dimensional magnetization prepared-rapid gradient echo; 3D FLAIR: three-dimensional fluid attenuated inversion recovery; 3D SWI: three-dimensional susceptibility-weighted imaging; DCE: dynamic-contrast enhancement; TR: repetition time; TE: time to echo; TI: inversion time; FOV: field of view; NEX: number of excitation; GRAPPA: GeneRalized autocalibrating partial parallel acquisition.
